# Health-related quality of life among Indian women with polycystic ovary syndrome: A cross-sectional study in Kolkata, West Bengal

**DOI:** 10.51866/oa.550

**Published:** 2025-03-04

**Authors:** Riya Halder, Rabindra Nath Sinha, Tulika Jha, Tapobrata Guha Ray

**Affiliations:** 1 MBBS, MD (Community Medicine), Senior Resident, ESI-PGIMSR, ESIC Medical College and ESIC Hospital, ODC, EZ, Joka, Kolkata,West Bengal, India. Email: riyahalder94@gmail.com; 2 MBBS, MD (Community Medicine), Professor, Department of Community Medicine, Kolkata, West Bengal, India.; 3 MBBS, M.S (Obstetrics and Gynaecology), Associate Professor, Department of Obstetrics and Gynaecology, West Bengal, India.; 4 MBBS, MD (Community Medicine), Associate Professor, Department of, Community Medicine, West Bengal, India.

**Keywords:** India, Mental health, Polycystic ovary syndrome, Quality of life

## Abstract

**Introduction:**

The unprecedented rise in the prevalence of polycystic ovary syndrome (PCOS) among women of reproductive age has raised concerns regarding their well-being. This study aimed to assess the quality of life (QoL) of women with PCOS and its associated factors in a tertiary hospital in Kolkata, India.

**Methods:**

A cross-sectional study was conducted from October 2020 to September 2022 at the outpatient clinic of a tertiary hospital in Kolkata. Quantitative data were collected from 189 women with PCOS using a pretested questionnaire and analysed using SPSS version 16.

**Results:**

Around 27.5% of the participants had an overall poor QoL. The psychological domain was the most affected domain, with 42.3% showing a poor QoL. A significant negative correlation was found between the psychological domain score and time since PCOS diagnosis (P=0.001). Multivariable logistic regression analysis showed that being married (adjusted odds ratio [AOR]=2.92) and having an unhealthy waist-hip ratio (AO R= 1.97) were significantly associated with a poor psychological QoL, whereas having an age of ≤24 years (AOR=2.56) and being in below middle socioeconomic class (AOR=2.08) were significantly associated with poor social and environmental QoL, respectively.

**Conclusion:**

PCOS affects all domains of quality of life. Psychological distress is an important comorbidity of PCOS as with any other reproductive and metabolic comorbidities, necessitating the need for mental health screening and treatment among affected women. Healthcare providers should focus on various lifestyle modifications along with drug therapy to prevent the emergence of PCOS as a public health problem.

## Introduction

Polycystic ovary syndrome (PCOS) is the most common endocrinological disorder in women of reproductive age,^1-3^ and its burden has been increasing globally since 1990.^4^ The World Health Organization (WHO) estimates that 8%–13% of reproductive-aged women are affected by the disorder, of whom up to 70% remain undiagnosed worldwide.^5-8^ India has witnessed an approximately 30% rise in the prevalence of PCOS in the last couple of years,^9^ ranking among the top five countries in the world with a high number of incident cases and long disability-adjusted life-years in 2019.^4^ The prevalence of PCOS is 11.3% among Indian adult women and 12.3% among adolescents.^10^

PCOS, first described in 1935 by Irving F. Stein and Michael L. Leventhal, is a multifaceted disease associated with a wide spectrum of clinical manifestations and adverse complications, affecting women of childbearing, adolescence and postmenopausal age.^11^ A number of clinical features such as changes in physical appearance (i.e. obesity, excessive body hair or acne), hormonal disturbances (i.e. hyperandrogenism), metabolic changes (i.e. insulin resistance), menstrual cycle disturbances (i.e. amenorrhea or oligomenorrhea), infertility and long-term complications (e.g. increased risk for diabetes) are associated with PCOS.^12^

PCOS may affect women in different aspects, such as their emotions, identity and quality of life (QoL). According to the WHO, QoL considers an individual’s perception of their position in life in the context of the culture and value systems in which they live and in relation to their goals, expectations, standards and concerns.^13^ The International Association of Quality Life Research describes health-related quality of life (HRQoL) as the functional effect of a medical condition and/or its consequent therapy upon a patient.^14^ Many of the symptoms of PCOS have been reported as important concerns to women, as they are often associated with low self-esteem, physical debilitation, imbalances in the psychosexual sphere and disruption of daily activities, driving affected women towards psychological and behavioural disorders such as bipolar disorder, sadness, negative emotions, frustration and eating disorder. A higher prevalence of depression and anxiety is also reported among women with PCOS than among their counterparts.^15^

As there is a dearth of published literature on the HRQoL of Indian women with PCOS, we undertook a hospital-based study to assess the QoL of women diagnosed with PCOS and identify its associated factors.

## Methods

### Study design, setting and participants

This retrospective cross-sectional study was conducted from October 2020 to September 2022 in the outpatient clinic of the gynaecology and obstetrics department (GOPD) of R.G. Kar Medical College and Hospital, Kolkata, West Bengal, India, which has a footfall of around 2000 patients per day. This medical college hospital caters tertiary-level healthcare to a large population of North Kolkata and adjoining districts of North 24 Parganas, Howrah and Hooghly. The GOPD runs 6 days a week. All patients with PCOS, diagnosed using the Rotterdam criteria^16^ at least 6 months before the date of data collection, attending the GOPD for treatment/follow-up were included in the study. Patients who did not provide informed written consent, patients who were aged below 18 years and did not provide written assent or whose parents/legally authorised representatives did not provide written consent, patients who were diagnosed with psychiatric illness, patients who had severe neurological disease or patients who were unwilling to participate in the study were excluded.

### Sample size determination

The sample size was calculated using Cochran’s formula.^17^ The standard normal variate was taken as 1.96 (5% type-I error), the standard deviation of the total QoL score in women with PCOS as 11.3, according to a similar study on PCOS in Iran,^18^ and the relative error of precision as 15%. Given a non-response allowance of 10%, the final estimated sample size was 189.

### Sampling design and technique

A complete enumeration method was followed to select study participants. Among the 6 working days of the GOPD, 2 days were selected randomly for data collection. All patients who attended the GOPD on those days, fulfilled the inclusion criteria and consented as applicable were included in the study.

### Data collection methods, study tools and parameters used

Data collection was conducted via medical record checking for clinical and anthropometric examination as well as face-to-face interviews. Anthropometric measurements such as height, weight, waist circumference and hip circumference were measured by the principal investigator using a stadiometer (Prestige), calibrated weighing scale (Model: SF-180B) and non-stretchable measuring tape, respectively.

- Height^19^: Participants were asked to remove footwear and headgear, if any, and stand on the footboard of the stadiometer with their feet together, heels against the backboard and knees straight, looking straight ahead facing the investigator. Participants’ eyes were ensured to be at the same level as their ears. Subsequently, the measuring arm of the stadiometer was gently pulled down onto the head of participants while they were asked to breathe in and stand tall. Height was read in centimetres at the exact point to the nearest millimetre and further converted into metres. Participants were then asked to step away from the measuring board.
- Weight^19^: Participants were first asked to remove footwear, any heavy overclothing, watch and mobile phone from their pocket. Thereafter, they were instructed to stand still on the weighing scale with one foot on each side of the scale and the arms by the side and face forward. Weight was then recorded in kilogrammes.
- Waist circumference^20^: Participants were made to stand straight behind a guard to maintain privacy. The smallest part of the waist just above the belly button was measured over light clothing using the non-stretchable measuring tape.
- Hip circumference^20^: For measuring hip circumference, participants were first asked to stand straight behind a guard. Thereafter, measurement was taken around the largest part of the buttocks over light clothing.
- Waist-hip ratio^20^: The WHO criteria for measuring waist-hip ratio were used. It was calculated by dividing the waist circumference by the hip circumference and classified as follows:
   o Healthy: <0.85
   o Unhealthy: ≥0.85
- Body mass index (BMI)^20^: BMI was measured as weight in kilogrammes divided by the square of height in metres (kg/m^2^). The WHO classification of BMI was used in our study:
   o Underweight: <18.5
   o Normal: 18.5-24.9
   o Overweight: 25-29.9
   o Obese: ≥30

A pretested structured questionnaire was used to collect information from participants. The questionnaire was divided into the following sections: 1) socioeconomic characteristics, 2) behavioural profile (physical activity status, dietary habit and addiction), 3) clinical profile (BMI, hirsutism, waist-hip ratio, acne, type of treatment received in the last visit, family history, time since diagnosis and comorbidity), 4) reproductive health profile (menstrual health, obstetric history and sexual health), 5) mental health profile and 6) QoL - the outcome variable of the study.

The socioeconomic status (SES) of an individual or a family is an important determinant of the health and overall QoL of the respective family members. There are certain standard scales used to determine the SES of a family. In our study, the Modified B.G. Prasad Scale^21^ was used. This scale is applicable for both urban and rural areas and takes into account the monthly per capita income in rupees for classifying families into five social classes:

- Class I (upper class): ≥Rs 7863
- Class II (upper middle class): Rs 3931-7862
- Class III (middle class): Rs 2359-3930
- Class IV (lower middle class): Rs 1179-2358
- Class V (lower class): <Rs 1179

Physical activity was assessed using the International Physical Activity Questionnaire Short Form,^22^ which consists of seven items. The results are reported both as continuous variables (MET min/week) and in categories:

- High physical activity: 7 or more days of any combination of walking and moderate- or vigorous-intensity activities, achieving a minimum total physical activity of at least 3000 MET min/week.
- Moderate physical activity: 5 or more days of any combination of walking and moderate- or vigorous-intensity activities, achieving a minimum total physical activity of at least 600 MET min/week.
- Low physical activity: Not meeting any criteria for either moderate or high physical activity.

Dietary habits referred to the kinds of food participants consumed. They were classified into vegetarian or non-vegetarian diets. Data on any history of addiction and type of product addicted to (e.g. tobacco [e.g. bidi or cigarette], smokeless tobacco [e.g. gutkha, guraku, khaini, pan masala, jarda or raw tobacco] or alcohol) were also collected.

Hirsutism was assessed using a pictorial hirsutism scoring sheet^23^ based upon the Ferriman-Gallwey scoring system. This sheet evaluated nine anatomic regions: moustache and beard, chest, breast areola, linea alba, upper back, lower back, thighs, inner sides of the femur and external genitalia. Each area was scored from 0 (‘no terminal hair growth’) to 4 (‘maximum hair growth’). A score of <8 indicated no hirsutism; 8-16, mild hirsutism; 17-24, moderate hirsutism; and >24, severe hirsutism.

The length of time from the diagnosis of PCOS is considered an important predictor variable of QoL. Thus, information on the length of time between the date of diagnosis and the date of data collection - known as the time since diagnosis - was also collected.

The presence of comorbidities may affect the ability of an individual to function and influence their survival. Comorbidities include coexisting or additional diseases with reference to an index condition. Data on the presence of comorbidities were also collected from medical records.

The mental health profile was assessed using the Patient Health Questionnaire 4,^24^ which consists of four items rated on a Likert scale, ranging from 0 to 3. The score is interpreted as follows:

- 0-2: No psychological distress
- 3-5: Mild psychological distress
- 6-8: Moderate psychological distress
- 9-12: Severe psychological distress
- Anxiety subscale: Sum of items 1 and 2 (score range, 0-6)
- Depression subscale: Sum of items 3 and 4 (score range, 0-6)

On each subscale, a score of 3 or greater is considered positive for screening. In this study, participants who were screened to have anxiety or depression were referred to the psychiatry outpatient clinic of our hospital for further management.

The QoL of participants was assessed using the WHO Quality of Life-BREF (WHOQOL-BREF).^25^ The first two questions from this 26-item scale evaluate the overall perception of QoL and overall perception of health. The remaining questions are divided into four domains: physical (seven items), psychological (six items), social (three items) and environmental (seven items). Each item is scored on a Likert scale, ranging from 1 (‘very poor/very dissatisfied/not at all/never’) to 5 (‘very good/very satisfied/an extreme amount/always’). Three items (i.e. Q3, Q4 and Q26) are scored reversely. The total score within each domain is used to calculate the domain score (raw score). In our study, the first transformation method was adopted, which converted the domain scores to range from 4 to 20, comparable with the WHOQOL-100. The second transformation method converted the domain scores to range from 0 to 100 so that comparisons between domains could be conducted. Higher scores in each domain correspond to a greater perceived QoL. Furthermore, in our study, the median score obtained in each domain was taken as the cutoff value for categorising the QoL. Participants who scored less than the median score of a domain were considered to have a poor QoL in that respective domain.

The reliability of all scales used in the study was checked based on Cronbach’s alpha coefficients, along with inter-item correlation coefficients. Face and content validity of the questionnaire were ascertained for conceptual adequacy, relevance, comprehensiveness and clarity of each item by public health experts. The questionnaire was prepared in consultation with public health specialists and gynaecologists (who were also the co-authors), as per the stated objectives of the study and based on the local context. To establish face validity, another subject expert reviewed the questionnaire, who confirmed that each item measured the concept it intended to measure. Content validation was performed using the content validity index by calculating the content validity ratio of Lawshe.^17^ Six experts participated in the validation process. The questionnaire was prepared in English and was translated into Bengali and Hindi. Thereafter, it was again back-translated into English by two experts in the three languages, not knowing the original questionnaire. The back-translated English version was compared with the original English version, and discrepancies were corrected, maintaining semantic equivalence.

### Statistical analysis

Quantitative data were analysed using SPSS (IBM Corp., Armonk, NY, USA; version 28). Normality of the data was checked using a histogram, the Kolmogorov-Smirnov test and the Shapiro-Wilk test, with the significance level set at <0.05. Appropriate descriptive statistics were applied to denote the outcome variables as well as the independent variables. Nonparametric tests, including the Mann-Whitney U test (for two groups), were used to determine the mean rank differences between different groups of independent variables. The relationship between two continuous variables (independent and dependent variables) was evaluated using correlation analysis. Spearman’s correlation coefficients (p) were used due to the skewed distribution of the data. The factors associated with a poor QoL were identified via univariate logistic regression analysis for the biologically plausible predictor variables separately for each domain of the WHOQOL-BREF, considering P-values of <0.05 as significant. The predictor variables found to be significant in the univariate analysis at P-values of <0.25 were checked for multicollinearity (variance inflation factor cut-off: >10) before including them in the final multivariable logistic model. Model fitness was checked using the Hosmer-Lemeshow test (P>0.05).

## Results

Of the 189 participants, 59.3% were female patients aged 15-24 years. The median age of the participants was 23 years {interquartile range (IQR)=20-26 years}. More than half (58%) were from urban areas and were Muslims (57.7%). Around 32.4% of the participants completed secondary education, and the median duration of schooling was 12 years. Approximately 29.6% belonged to class III under the 2021 Modified B.G. Prasad Scale. The majority of the participants (81.5%) were unemployed, and more than half (62%) were married, among whom 60.3% were married for 1-5 years (Table 1).

**Table 1:** Sociodemographic characteristics of the participants (N=189).

Parameter	Category	Number (%)
Age (year)	<15	2 (1.0)
	15-24	112 (59.3)
	25-34	73 (38.6)
	35-49	2 (1.1)
Residence	Urban	110 (58.2)
	Rural	79 (41.8)
Religion	Hinduism	79 (41.8)
	Islam	109 (57.7)
	Christianity	1 (0.5)
Educational level^@^	Non-formal education	1 (0.5)
	Pre-primary education	7 (3.7)
	Primary education	28 (14.8)
	Upper primary education	42 (22.2)
	Secondary education	61 (32.4)
	Senior secondary education and above	50 (26.4)
Socioeconomic status^$^	Class I (upper class)	23 (12.2)
	Class II (upper middle class)	40 (21.2)
	Class III (middle class)	56 (29.6)
	Class IV (lower middle class)	40 (21.1)
	Class V (lower class)	30 (15.9)
Employment status	Employed	36 (18.5)
	Unemployed	153 (81.5)
Marital status	Never married	70 (37.0)
	Married	116 (61.4)
	Divorced	3 (1.6)
Duration of marriage (year)	<1	3 (2.6)
	1-5	70 (60.3)
	6-10	31 (26.7)
	>10	12 (10.4)

@ Non-formal education: Any organised systematic generally intentional and planned educational activity carried outside the framework of the formal system, pre-primary education: below 5th standard, primary education: completed class V, upper primary education: completed class VIII, secondary education: passed class X, senior secondary education and above: passed class XII.

$ According to the 2021 Modified B.G. Prasad Scale

Around 54.5% of the participants did moderate physical activity (600-2999 MET min/week), and 97.3% followed a non-vegetarian diet. Only few participants reported addiction. Approximately 2.1% were ever addicted to some substance.

The BMI of the participants ranged from 15.4 to 39.4 kg/m^2^, with a median value of 24.3 kg/m^2^. Half of the participants (50.2%) had normal weight, while 28.6% were overweight. Around 35.9% had acne; 50.8%, hirsutism; and 59.8%, increased waist-hip ratio. The majority of the participants (75.1%) had no family history of a similar health problem. More than half (64.0%) were put on pharmacological treatment only; 30.7% were advised for lifestyle modification; and only 5.3% were on both pharmacologic and non-pharmacologic treatments. Approximately 31.7% presented with at least one comorbidity, the majority of whom had hypothyroidism (73.3%). Female patients diagnosed with PCOS for at least 6 months were included in our study. Around half of them (50.3%) were diagnosed for less than 1 year (Table 2).

**Table 2:** Behavioural and clinical profiles of the participants (N=189).

Parameter	Category	Number (%)
Physical activity done in the last 7 days	High (≥3000 MET min/week)	23 (12.2)
	Moderate (600-2999 MET min/week)	103 (54.5)
	Low (<600 MET min/week)	63 (33.3)
Type of diet	Vegetarian	5 (2.7)
	Non-vegetarian	184 (97.3)
Addiction	Absent	185 (97.9)
	Present^@^	4 (2.1)
BMI (WHO classification)	Underweight (<18.5 kg/m^2^)	15 (7.9)
	Normal (18.5–24.9 kg/m^2^)	95 (50.2)
	Overweight (25–29.9 kg/m^2^)	54 (28.6)
	Obese (≥30 kg/m^2^)	25 (13.3)
Acne	Present	68 (36.0)
	Absent	121 (64.0)
Hirsutism (score)	Absent (<8)	93 (49.2)
	Mild (8–16)	83 (43.9)
	Moderate (17–24)	13 (6.9)
Waist–hip ratio (WHO criteria)	Healthy (<0.85)	76 (40.2)
	Unhealthy (≥0.85)	113 (59.8)
Family history	Present	47 (24.9)
	Absent	142 (75.1)
Type of treatment received in the last visit	Lifestyle modification^$^	58 (30.7)
	Pharmacologic treatment	121 (64.0)
	Pharmacologic treatment + lifestyle modification	10 (5.3)
Comorbidities	Absent	129 (68.3)
	Present^*^	60 (31.7)
Time since diagnosis^#^	6 months to 1 year	95 (50.3)
	1–5 years	78 (41.2)
	>5 years	16 (8.5)

@ Around three women ever used smokeless tobacco (guraku and gutkha), and one woman used to drink alcohol

$ Lifestyle modification: Dietary change and physical activity

* Multiple responses: Comorbidities included hypothyroidism, migraine, hypertension, type 2 diabetes mellitus, asthma, fatty liver disease, pituitary microadenoma, haemorrhagic ovarian cyst and inflammatory bowel disease

# Length of time between the date of diagnosis and the date of data collection

The median age at menarche of the participants was 13 years (IQR=12–14 years). The median length of the menstrual cycle was 60 days (IQR=30–82 days). Approximately 60.3% of the participants had oligomenorrhea, whereas 38.1% had menorrhagia. Among the 119 ever-married participants, 72.3% did not have any living children, while 77.4% were trying to conceive up to 2 years. Primary infertility was found in 34.9% of the participants.

Among the participants, 82.5% had some form of psychological distress. More than half (52.4%) were screened to have depression, and 45% experienced anxiety.

### QoL

**Figure 1:**
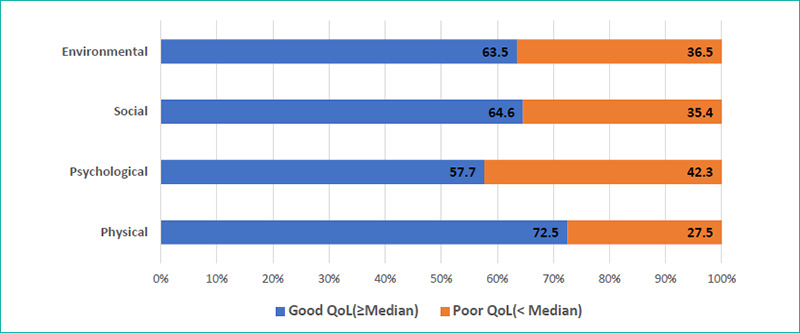
Proportional bar diagram showing the distribution of the participants according to their quality of life in different domains (N=189).

**Figure 2:**
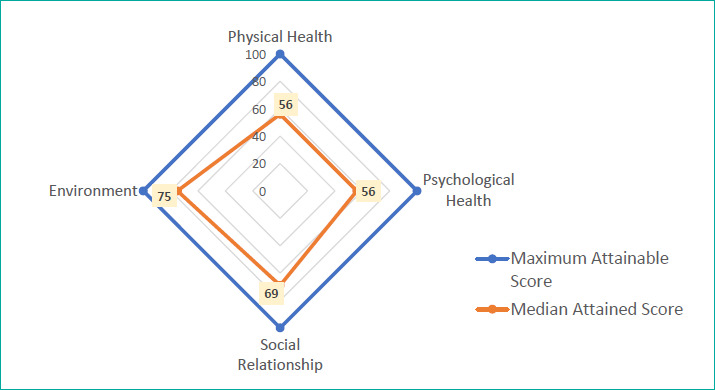
Radar chart showing the median transformed scores obtained in each domain of the WHOQOL-BREF (N=189).

Around 27.5% of the participants rated their overall QoL as poor to very poor, whereas 39.7% were dissatisfied or very dissatisfied with their overall health status. Additionally, 42.3% had a poor psychological QoL (42.3%) (Figure 1). The median WHOQOL-BREF score was 75 (IQR=69–88) for the environmental domain, 69 (IQR=50–81) for the social relationship domain, 56 (IQR=50–69) for the physical domain and 56 (IQR=44–69) for the psychological domain (Figure 2).

Increasing BMI (p=-0.712, P=0.04) and red meat intake (p=-0.163, P=0.03) were found to be negatively correlated with the physical domain score. Further, a significant negative correlation was observed between the psychological domain score and time since PCOS diagnosis (p=-0.233, P=0.001).

### Factors associated with QoL

The significant factors associated with a poor psychological QoL among die participants were being married (adjusted odds ratio [AOR]=2.92, 95% confidence interval [CI] = 1.52-5.59) and having an increased waist-hip ratio (AOR=1.97, 95% CI=1.04-3.72). An age of ≤24 years (AOR=2.56, 95% CI=1.32-4.95) and an SES of below middle class (AOR=2.08, 95% CI=L09-3.99) were found to be significantly associatad with poor social and environmental QoL_l_ respectively. Since no significant factors emerged from the univariate regression analysis of the factass assogiated with a poor physical QoL, a multivariable regression mydel gould not be foonoulated (Table 3).

**Table 3:** Multivariable logistic regression analonis of the factops associated with a poor psychological, social and environmental quality of life of the participants (N=189).

Parameter	Total number	Poor QoL (%)	Unadjusted OR (95% CI)	P-value	Adjusted OR (95% CI)	P-value
***Psychological domain***						
*Marital status*						
Married	116	59 (50.9)	2.56 (1.37-4.78)	0.003	2.92 (1.52-5.59)	**0.001**
Unmarried	73	21 (28.8)	1		1	
*Waist–hip ratio*						
Healthy	76	26 (34.2)	1		1	
Unhealthy	113	54 (47.8)	1.76 (0.96-3.20)	0.06	1.97 (1.04-3.72)	**0.03**
Duration from diagnosis (↑)^*^			0.98 (0.97-1.00)	0.06	0.98 (0.97-1.00)	0.12
*Comorbidity*						
Absent	129	59 (45.7)	1		1	
Present	60	21 (35.0)	0.63 (0.33-1.20)	0.16	0.57 (0.29-1.12)	0.10
***Social domain***						
*Age*						
≤24 years	114	49 (43.0)	2.38 (1.25-4.55)	0.008	2.56 (1.32-4.95)	**0.005**
>24 years	75	18 (24.0)	1		1	
Psychological distress (↑)^*^			1.08 (0.98-1.20)	0.10	1.10 (0.99-1.22)	0.057
***Environmental domain***						
*Residence*						
Urban	79	33 (41.8)	1		1	
Rural	110	36 (32.7)	1.47 (0.81-2.68)	0.20	1.14 (0.60-2.16)	0.68
*Religion*						
Muslim	109	34 (31.1)	0.60 (0.33-1.09)	0.09	0.57 (0.30-1.00)	0.07
Hindu + Christian	80	35 (43.7)	1		1	
*Socioeconomic status* ^#^						
Middle class and above	119	36 (30.3)	1		1	
Below middle class	70	33 (47.1)	2.05 (1.11-3.78)	0.02	2.08 (1.09-3.99)	0.02

* OR: odds ratio, CI: confidence interval, Continuous variables

# According to the 2021 Modified B.G. Prasad Scale

Fit and variance of the logistic regression model for psychological quality of life: Hosmer-Lemeshow test statistic=3.247, P-value=0.918 Cox and Snell’s R2=0.189, Nagelkerke’s R2=0.213 Fit and variance of the logistic regression model for social quality of life: Hosmer-Lemeshow test statistic=2.90, P-value=0.940 Cox and Snell’s R2=0.157, Nagelkerke’s R2=0.178 Fit and variance of the logistic regression model for environmental quality of life: Hosmer-Lemeshow test statistic=16.04, P-value=0.10 Cox and Snell’s R2=0.204, Nagelkerke’s R2=0.263

## Discussion

This study revealed that 27.5% of the participants had an overall poor QoL, and 39.7% were dissatisfied with their overall health status. Around 42.3% had a poor psychological QoL, similar to the findings of Korampatta et al.,^26^ wherein the psychological health of 74% of their participants was significantly affected. In Udaipur, India, Saxena et al.^27^ also found that the psychological QoL of affected patients received the lowest score, thereby proving that patients with psychiatric morbidity have a significantly lower QoL. In our study, the maximum median score was obtained in the environmental domain (75), followed by the social relationship domain (69), physical domain (56) and psychological domain (56). A study conducted in Karnataka, India, reported median scores of 44, 56, 69 and 75 in the physical, psychological, social relationship and environmental domains, respectively, using the same WHOQOL-BREF.^28^ In India, D’Souza et al. similarly obtained mean scores of 20.07, 16.85, 18.17 and 40 in the physical, psychological, social relationship and environmental domains, respectively.^29^ These findings are quite similar to those of our study, where the highest score was obtained in the environmental domain.

Being married showed a significant association with a poor psychological QoL among our participants. One of the major complications of PCOS is infertility. An inability to conceive often leads to marital disputes within the family, which can degrade the mental health status of not only the woman but also her family members. A recent qualitative study conducted in Iran^30^ concluded that a woman with infertility often feels inferior and less of a woman. A strong association between infertility and the emotional well-being of patients with PCOS was found in the study performed in Patna, India, by Tabassum et al.^31^ Fertility treatment for PCOS can involve emotional, ethical, moral and even religious dilemmas.

An increased waist-hip ratio was another factor significantly associated with a poor psychological QoL among our participants. This may be attributed to the fact that PCOS frequently manifests itself at puberty or during young adulthood with features such as obesity. Obesity has been reported as an important concern to women with PCOS, especially in adolescents, as it is often associated with low self-esteem due to negative body image.^32^

In the present study, the female patients aged less than 24 years showed a poor social QoL owing to the development of self-identity and body image concerns.^33^ Changes in outer appearance form an important area of concern for these young women, as they influence their social QoL. In their study conducted in Pakistan, Malik et al.^34^ revealed that emotions were better managed among women with PCOS in older age groups (i.e. 36-50 years). These findings show that age plays a significant role in the emotional well-being of patients with PCOS.

The participants belonging to below-middle-class families showed a poor environmental QoL. This is in line with the findings from the study performed by Rzonca et al.^35^ in Poland, which demonstrated that the overall QoL improved with better socioeconomic standing of participants.

## Conclusion

PCOS, the commonest hormonal disorder among women, adversely affects all domains of QoL, especially the psychological domain. Psychological distress forms a common and important comorbidity of PCOS necessitating the need for screening and appropriate management of the mental health conditions of affected women. Women with PCOS have to cope with different types of physical, mental and social challenges every day. This study shows a strong need for addressing the mental health aspects of these patients. PCOS should no longer be considered a medical problem for women alone. The time has come to consider it as a unique and important public health problem of the country.

## Data sharing statement

Raw data are available upon request to the corresponding author.

**How does this paper make a difference in general practice?**

- The findings of this study compel a new perspective on the commonest hormonal disorder in women – polycystic ovary syndrome. It is essential to prioritise the mental well-being of women affected by this condition, alongside addressing their reproductive health, to improve their overall quality of life.
